# Incarcerated prolapsed ureterocele masquerading as a vulvar mass after midurethral sling surgery: a case report with systematic review

**DOI:** 10.3389/fsurg.2026.1773513

**Published:** 2026-06-09

**Authors:** Hongyun Zhang, Jiang Zhu, Zihan Zhou, Xia Tao, Liqun Sun, Hong Zhan

**Affiliations:** 1 Department of Ultrasound, Women’s Hospital, Zhejiang University School of Medicine, Hangzhou, China; 2 Zhejiang University School of Medicine, Hangzhou, China; 3 Day Surgery Center, Women’s Hospital, Zhejiang University School of Medicine, Hangzhou, China; 4 Zhejiang Provincial Key Laboratory of Precision Diagnosis and Therapy for Major Gynecological Diseases, Women’s Hospital, Zhejiang University School of Medicine, Hangzhou, China

**Keywords:** midurethral slings, pelvic organ prolapse, sonovaginography, ultrasonography, ureterocele

## Abstract

**Background:**

Incarcerated prolapsed ureterocele (IPU) after midurethral sling (MUS) surgery is extremely rare and may be misdiagnosed as a vulvar or interlabial mass because of its atypical presentation and altered postoperative anatomy.

**Methods:**

We report the clinical presentation, multimodal ultrasonographic findings, surgical management, and follow-up of a patient with IPU after MUS surgery. A systematic review was conducted in accordance with the PRISMA framework using major medical databases to identify previously reported cases. Fifteen cases were identified from the literature; together with the present case, 16 cases were included in the analysis.

**Results:**

The patient presented with a vulvar mass after MUS surgery, and the diagnosis was difficult because of altered postoperative pelvic anatomy. Multimodal ultrasonography demonstrated a characteristic dumbbell sign, showing continuity between the intravesical and prolapsed components, and dynamically clarified the relationship between the prolapsed ureterocele and the sling in the postoperative setting. In the 16 included cases, prolapsed ureterocele was an uncommon but important differential diagnosis in women presenting with an interlabial or vulvar mass, especially when accompanied by urinary symptoms. Imaging was essential for diagnosis, and surgical treatment was generally associated with favorable outcomes.

**Conclusion:**

IPU after MUS surgery is a rare but clinically important condition that should be considered in the differential diagnosis of postoperative vulvar or interlabial masses. Multimodal ultrasonography can provide dynamic assessment of ureterocele anatomy and its relationship to the sling, while CT and MRI remain useful complementary modalities in selected cases.

## 1 Introduction

Ureteroceles are predominantly congenital anomalies arising from aberrant embryological development. Specifically, the failure of complete resorption and degeneration of the membrane between the ureter and the urogenital sinus results in ureteral orifice stenosis or maldevelopment of the distal ureteral wall, culminating in cystic dilation. Prolapsed ureterocele typically arises in association with an ectopic ureteral orifice of a duplicated renal collecting system and demonstrates a higher prevalence in female fetuses (1, 2). Ureteroceles are uncommon in adolescents and adults (3). Even more infrequent is the presentation of a prolapsed ureterocele, particularly in the context of prior urological intervention.

Stress urinary incontinence (SUI), defined as the involuntary leakage of urine during activities that increase intra-abdominal pressure—such as coughing, sneezing, or exertion—has a reported prevalence of approximately 46% (4). The underlying pathophysiology generally implicates urethral hypermobility or intrinsic sphincter deficiency. In exceedingly rare cases, however, mechanical obstruction or disruption of urethral closure by a ureterocele may mimic or contribute to SUI symptoms (5). The midurethral sling (MUS) procedure remains a mainstay in the surgical management of SUI, with its safety and therapeutic efficacy well-established. Nonetheless, postoperative complications—though infrequent—can be significant (6). Among these, prolapse of a ureterocele with incarceration constitutes a rare but serious event that may precipitate acute urinary tract obstruction and, in severe cases, compromise renal function, thereby exerting a profound impact on patient well-being (7). In this setting, accurate imaging evaluation is essential. Multimodal ultrasound, including transrectal biplane high-frequency ultrasound (TBHU) and sonovaginography (SVG), may help delineate the relationship between the ureterocele, urethra, and sling. This approach can also demonstrate a dumbbell-like configuration, which we describe in this report as the “dumbbell sign”: the intravesical and prolapsed extravesical components of the ureterocele connected through a narrowed segment. However, its use in gynecologic practice has been only rarely reported.

Here, we report a case of incarcerated prolapsed ureterocele after MUS surgery, which was initially misdiagnosed as a vulvar cyst and was subsequently diagnosed by multimodal ultrasound and confirmed by cystoscopy. We also reviewed 15 previously reported cases; together with the present case, 16 adult cases were included in the analysis. This study summarizes the clinical and imaging features of this rare condition, with particular attention to the diagnostic value of TBHU and SVG. The present case is notable for the postoperative diagnostic challenge after MUS surgery and for the use of combined TBHU and SVG to demonstrate the dumbbell sign and clarify the relationship between the prolapsed ureterocele, urethra, and sling.

## 2 Case presentation

A 37-year-old woman with a one-year history of SUI and dysuria underwent a MUS procedure at a local medical facility. Postoperatively, she experienced no improvement in urinary urgency and frequency, with increased voiding episodes. One month after surgery, she developed a progressively enlarging vulvar mass, worsening dysuria, suprapubic pressure, lower abdominal distension during micturition, and lumbosacral pain. Voiding required manual compression of the vulvar mass. The mass remained irreducible despite initial interventions, and persistent SUI significantly impaired her quality of life. She was referred to the Women's Hospital, Zhejiang University School of Medicine, for further evaluation in December 2024. Because the MUS procedure had been performed at an outside hospital, complete preoperative records were unavailable. Dedicated urinary tract imaging before MUS surgery could not be retrieved; therefore, it remained unclear whether the ureterocele had been recognized preoperatively.

**Figure 1:**
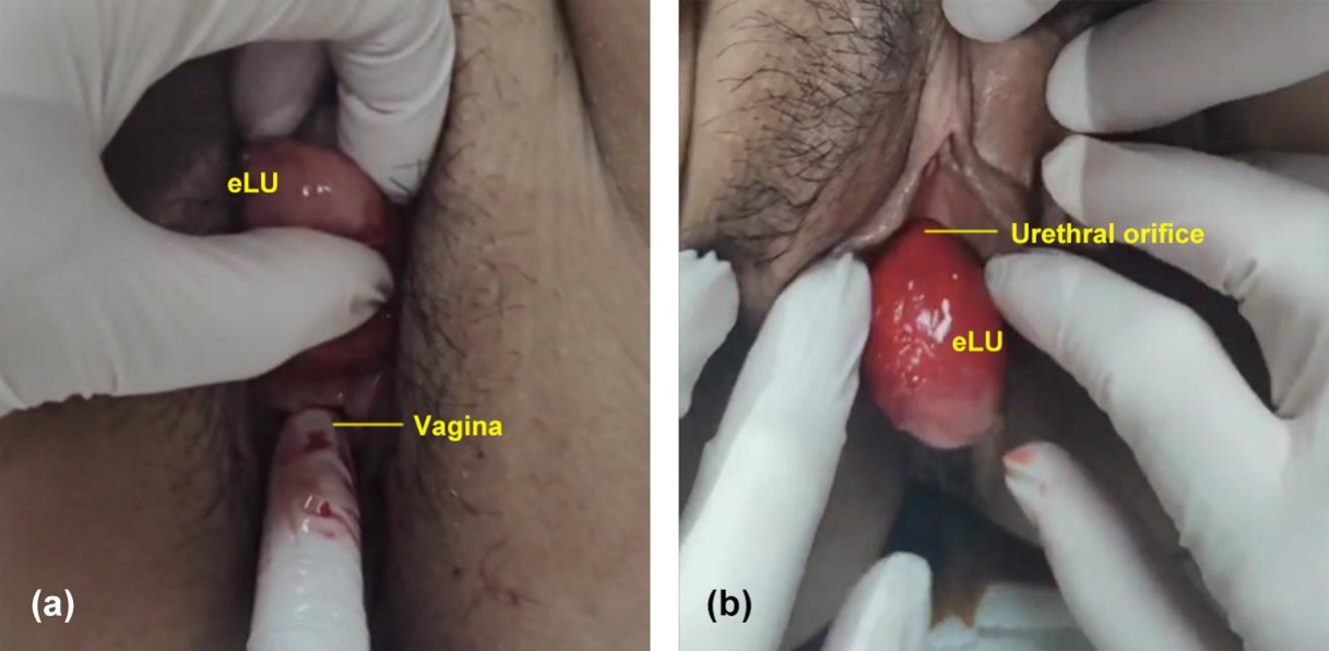
Incarcerated prolapsed ureterocele following midurethral sling. **(a,b)** In the present case, external view showing a vulvar cyst, 7 × 6 cm. in size, which was confirmed to be the prolapsed left ureterocele (LU). **(a)** The cavity that the finger entered below the mass is the vagina. **(b)** the ureterocele prolapsing beyond the urethral orifice.

**Figure 2:**
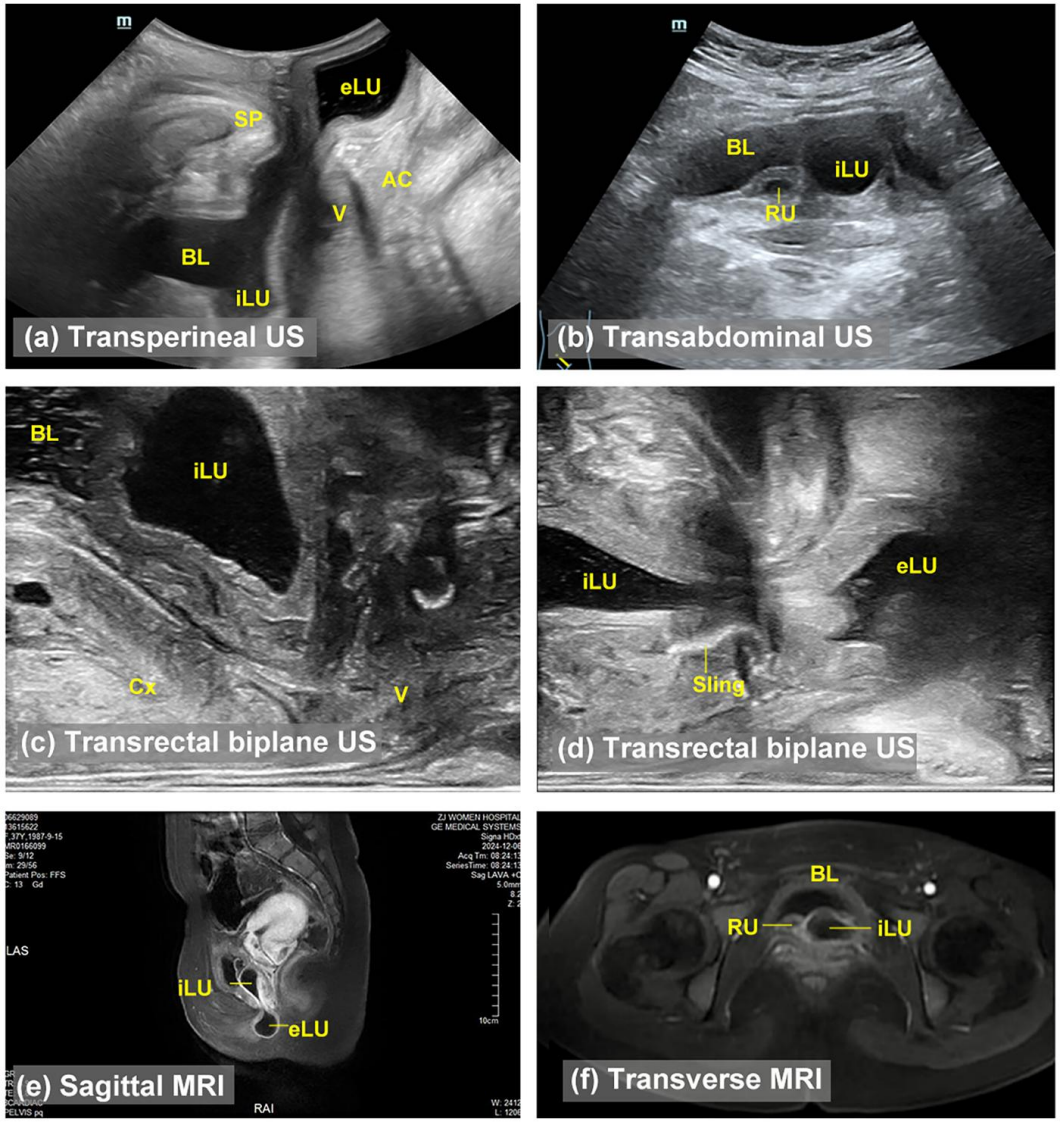
Multimodal imaging of an incarcerated prolapsed ureterocele in case 1. **(a)** Transperineal ultrasonography (US) demonstrating a dumbbell-shaped ureterocele, with a connection between the intra-bladder (iLU) and extra-bladder (eLU) components. **(b)** Transabdominal US revealing bilateral ureteroceles—left (iLU) and right (RU)—within the bladder trigone. **(c)** Transrectal biplane high-frequency ultrasound (TBHU) confirming partial location of the iLU within the bladder. **(d)** TBHU illustrating the sling echo within the anterior vaginal wall and mid-urethra, corresponding to urethral constriction; the sling divides the LU into iLU and eLU, forming a dumbbell-like configuration. **(e,f)** Pelvic magnetic resonance imaging (MRI) demonstrated that the mass originated from the urinary tract and contained fluid. **(e)** T1-weighted sagittal MRI of the pelvis demonstrating the fluid-filled ureterocele as a dumbbell, with clear separation into iLU and eLU. **(f)** T2-weighted transverse MRI at the bladder level confirming the intravesical location of the iLU. SP, symphysis pubis; BL, bladder; V, vagina; Cx, Cervix; AC, anal canal; LU, left ureterocele; RU, Right ureterocele; iLU, the intra-bladder part of the left ureterocele; eLU, the extra-bladder part of the left ureterocele.

On examination, an egg-sized mass was noted at the external urethral orifice: the anterior surface appeared pink and smooth, while the remainder was erythematous and granular, without contact bleeding (Figures 1a,b). Two-dimensional ultrasonography revealed bilateral single renal pelvises and dilation of the left ureter. Multimodal ultrasonography further showed that a portion of the left ureterocele (LU, 7.2 × 3.1 cm) protruded outside the bladder (eLU), while another portion remained inside (iLU) (Figure 2a).

Transabdominal ultrasonography showed the LU and right ureterocele (RU) at the bladder trigone (Figure 2b). Upon TBHU-guided reduction of the prolapsed component, the intravesical portion of the left ureterocele (iLU) was visualized as a cystic lesion (4.2 × 2.5 cm) at the left bladder trigone (Figure 2c). An echogenic sling mesh was evident in the anterior vaginal wall and midurethra, corresponding to narrowing of the intraurethral cystic segment (Figure 2d). T1-weighted sagittal MRI confirmed a bilobed configuration of the LU, comprising intravesical and extravesical components (Figure 2e). T2-weighted transverse MRI at the bladder level confirmed the intravesical location of the iLU and RU, corroborating the ultrasonographic findings (Figure 2f). The characteristic dumbbell-shaped appearance connecting the iLU and eLU was detected by both ultrasonography (Figures 2a,d) and MRI (Figure 2e). Cystoscopic evaluation confirmed bilateral ureteroceles. Surgical management included resection of the left ureterocele, placement of a left ureteral stent, anterior vaginal wall repair, and distal repositioning (approximately 1 cm) of the incised midportion of the MUS mesh. Postoperative recovery was uneventful. At one- and three-month follow-ups, the patient remained asymptomatic, with no recurrence of urgency, frequency, or urethral prolapse.

## 3 Systematic review methods

We searched PubMed, Embase, Scopus, and Web of Science from inception to February 2026 using combinations of the terms “prolapsed ureterocele,” “incarcerated ureterocele,” “adult,” “female,” and related synonyms. No language restrictions were applied, and the reference lists of included articles were also screened manually to identify additional eligible reports. The review aimed to identify published reports of prolapsed or incarcerated ureterocele in adult women, with particular attention to cases occurring after MUS surgery or presenting as an interlabial or vulvar mass.

Inclusion criteria were: (1) adult female patients; (2) prolapsed or incarcerated ureterocele confirmed by imaging, cystoscopy, or surgery; and (3) sufficient clinical detail on presentation, diagnosis, treatment, or outcome. Exclusion criteria were as follows: pediatric cases, male cases, animal cases, review articles, and reports with insufficient diagnostic information.

**Figure 3:**
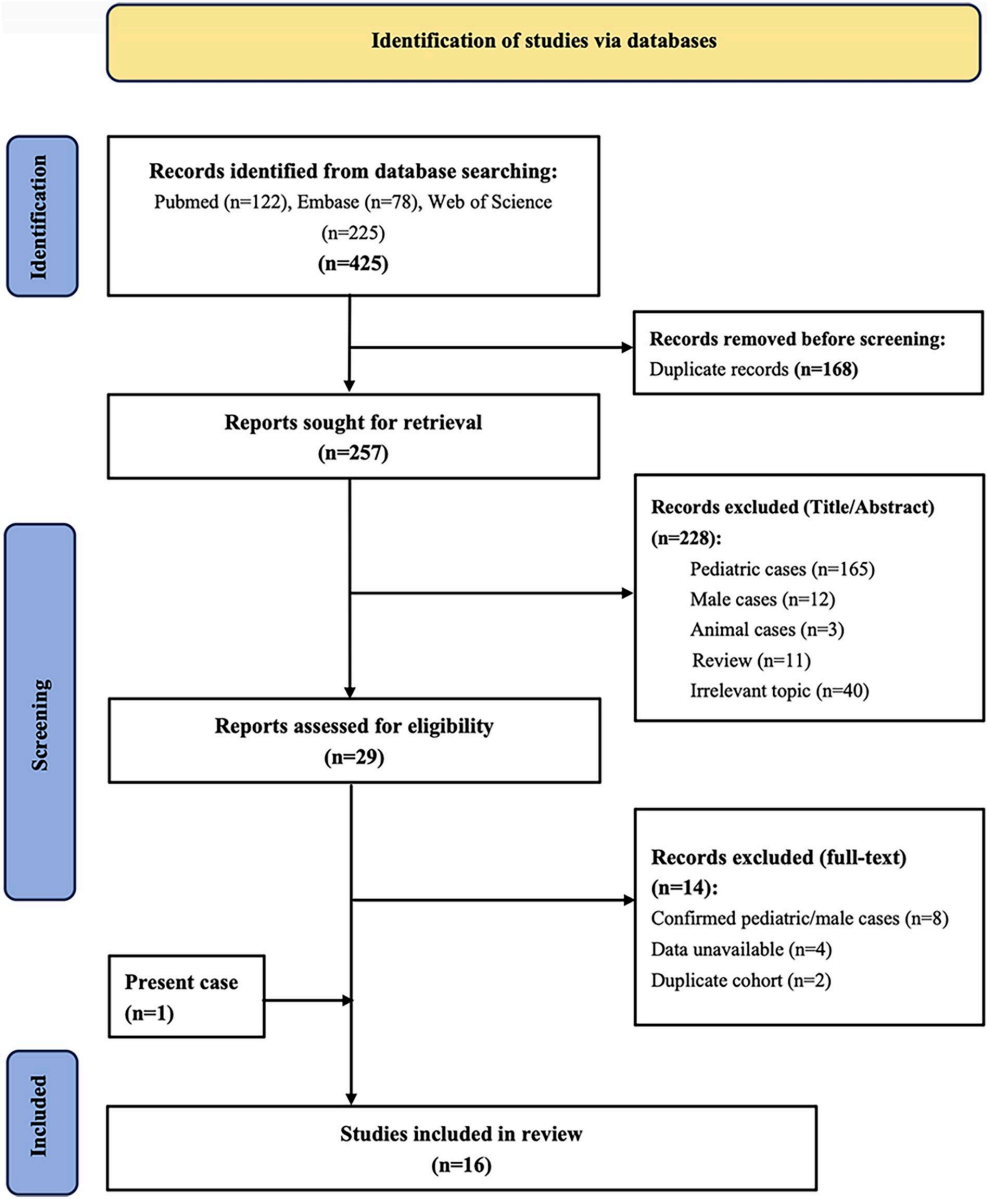
The PRISMA flow diagram.

Two reviewers independently screened titles/abstracts, followed by full-text assessment for eligibility. Disagreements were resolved by discussion. Study selection is summarized in the PRISMA flow diagram (Figure 3). Data were extracted independently by two reviewers using a standardized form, including age, symptoms, imaging findings, associated anomalies, treatment, and outcomes. The reporting completeness of the included case reports was appraised using the Joanna Briggs Institute checklist for case reports (Supplementary Table S1). This appraisal was not used to generate an overall risk-of-bias category. Because all included studies were case reports, pooled descriptive statistics were used only for descriptive and exploratory purposes.

## 4 Results of literature review

The present case was included as Patient 1 (P1) in the pooled analysis. Fifteen cases were identified from the literature; together with the present case, 16 cases were included in the analysis (Table 1). The mean age at presentation was 39.44 ± 12.97 years, encompassing both nulliparous and multiparous women (up to three previous births). Most cases presented with a vulvar or interlabial mass as the primary complaint. Common clinical manifestations included dysuria (56.25%, 9/16), urinary incontinence (25%, 4/16), and urolithiasis (18.75%, 3/16) [3,8,11]. Patients with urolithiasis exhibited either isolated dysuria (8, 9) or remained asymptomatic (10). Additional presentations included intense urethral bleeding (11) and hydronephrosis with proximal ureteral dilation (3). Notably, 18.75% (3/16) had prior MUS surgery. Among the four urinary incontinence cases, the present case and two published cases occurred after MUS surgery (7, 12), whereas one published case was reported without prior MUS surgery (13). Duplicated urinary tract systems were reported in 25% (4/16) of cases (14–17). Dysuria was also the predominant symptom in several other reports (18–20). Among the 15 cases with available measurements, the mean ureterocele diameter was 5.21 ± 2.06 cm. Imaging modalities included ultrasound (50%, 8/16), MRI (25%, 4/16), CT (18.75%, 3/16), and intravenous pyelography (IVP) (31.25%, 5/16); several patients underwent more than one imaging examination. Management involved cystoscopic intervention (68.75%, 11/16) or open surgery (31.25%, 5/16). Overall, the JBI appraisal suggested generally good reporting completeness, although several reports lacked detailed timelines, follow-up information, or explicit reporting of adverse events. These findings show that prolapsed ureterocele in adult women is rare and clinically heterogeneous, with imaging playing an important role in diagnosis and treatment planning.

**Table 1:** Prolapsed ureterocele in adult females: case summary.

Patients	Age (years)	Obstetric history	Stress urinary incontinence	Dysuria	Urinary calculi	Midurethral sling	Renal and ureteral duplication	Diameter of ureteroceles (cm)	Imaging examination	Surgical approaches
P1	37	G2P2	Yes	**Yes**	No	**Yes**	No	7	US, MRI	Cystoscopy
P2 (17)	24	NR	No	No	No	No	**Yes**	7	US, IVP	Cystoscopy
P3 (15)	54	NR^a^	No	**Yes**	No	No	**Yes**	3	US, IVP	Cystoscopy
P4 (16)	19	G0P0	No	**Yes**	No	No	**Yes**	5	CT	Cystoscopy
P5 (3)	22	G0P0	No	No	No	No	No	4	US	Open surgery
P6 (20)	26	G0P0	No	**Yes**	No	No	No	6	MRI	Cystoscopy
P7 (11)	45	NR	No	No	No	No	No	4.7	US	Cystoscopy
P8 (7)	50	NR	**Yes**	No	No	**Yes**	No	10	MRI	Cystoscopy
P9 (13)	44	NR	**Yes**	No	No	No	No	7	CT	Cystoscopy
P10 (8)	35	NR	No	**Yes**	**Yes**	No	No	5	x-ray, US, IVP	Open surgery
P11 (10)	29	G1P1	No	No	**Yes**	No	No	4	US	Open surgery
P12 (12)	41	G3P3	**Yes**	No	No	**Yes**	No	2	CT	Cystoscopy
P13 (19)	55	NR	No	**Yes**	No	No	No	NR	US, MRI	Cystoscopy
P14 (18)	48	NR	No	**Yes**	No	No	No	5	IVP	Cystoscopy
P15 (14)	39	NR	No	**Yes**	No	No	**Yes**	6	NR	Open surgery
P16 (9)	63	NR	No	**Yes**	**Yes**	No	No	2.5	IVP	Open surgery

a NR, not reported; MRI, magnetic resonance; CT, computed tomography; US, ultrasonography; IVP, intravenous pyelography.

Bold values indicate “Yes” responses and are used to highlight the presence of the corresponding clinical feature or management factor.

## 5 Integrated discussion

### 5.1 Prolapsed ureterocele in adult women and possible relationship with MUS surgery

Ureterocele is a congenital anomaly characterized by cystic dilatation of the distal ureter, frequently resulting in partial obstruction of urine flow at the ureterovesical junction. The prevalence in females is estimated to range from 1 in 5,000 to 1 in 12,000 individuals (3). Although most commonly diagnosed in pediatric populations, ureteroceles can also present in adults, where the clinical features tend to diverge significantly from those observed in children. In adult women, ureteroceles are often associated with recurrent urinary tract infections, stone formation, or, more rarely, prolapse. A prolapsed ureterocele refers to the downward herniation of the ureterocele into the urethra or even beyond the urethral meatus, potentially presenting as an external perineal or vulvar mass. This presentation can mimic other pelvic masses and lead to diagnostic challenges. It may also result in functional urinary obstruction, requiring a high index of suspicion and thorough differential diagnosis.

The pathophysiology underlying prolapsed ureterocele in adult females is multifactorial and remains incompletely understood. Ureterocele arises from congenital malformation, primarily due to a defect in the incorporation of the ureter into the bladder, leading to distal ureteral dilatation (16). In adults, additional contributing factors may include pelvic organ prolapse (POP) and age-related degeneration of pelvic support structures. It has been hypothesized that the weakening of pelvic connective tissues—common in women with POP—may exacerbate the descent and prolapse of an existing ureterocele (21). Furthermore, embryological remnants such as the Chwalla membrane, which plays a role in the normal development of the ureterovesical junction, may fail to resolve during fetal development, leading to the formation of a ureterocele (22).

Hormonal changes in postmenopausal women may also influence pelvic connective tissue and muscular integrity, increasing susceptibility to ureterocele prolapse. Anatomical anomalies, such as duplicated urinary collecting systems, are frequently associated with ureteroceles and may further complicate their presentation and management. The relationship between prolapsed ureterocele and SUI is complex and reflects interactions between anatomical disruption and functional bladder outlet obstruction. Prolapsed ureteroceles can present with acute urinary retention, a reducible vulvar mass, and symptoms of incomplete bladder emptying or intermittent voiding. These conditions contribute to functional incontinence by creating transient increases in intravesical pressure and outlet obstruction (21). In some instances, the ureterocele may mimic a vulvar mass or be associated with SUI through mechanical obstruction, mucosal irritation, or direct compression of the bladder neck (16, 23, 24).

Emerging evidence suggests a broader association between prolapsed ureterocele and pelvic floor dysfunction. A study evaluating the correlation between POP and lower urinary tract symptoms demonstrated that prolapse—including ureterocele—can exacerbate SUI and overactive bladder symptoms (25). Consequently, successful management of prolapsed ureterocele requires an approach that accounts for both anatomical abnormalities and functional disturbances of the lower urinary tract (26).

This case, contextualized within a pooled analysis of 16 adult female cases, raises the possibility that MUS procedures—although effective for stress urinary incontinence—may be associated with prolapse or incarceration of a previously unrecognized ureterocele in selected patients. The proposed mechanism is mechanical narrowing at the midurethra produced by the sling, which may impede spontaneous reduction of the prolapsed distal ureterocele during straining. The dumbbell sign can be understood anatomically as two communicating components of the same lesion: an intravesical cystic segment and a prolapsed distal segment, connected across the urethral outlet where the sling and periurethral tissues create a zone of constriction. This configuration may explain why the lesion appears bilobed on multimodal ultrasonography and why incarceration may occur after MUS surgery.

### 5.2 Diagnostic approach and the role of multimodal ultrasonography

The diagnosis of a prolapsed ureterocele in adult females poses a significant clinical challenge due to its rarity and the diversity of presenting symptoms. While some ureteroceles are identified incidentally during imaging studies for unrelated conditions, others manifest with urinary tract infections, urinary obstruction, or the appearance of a protruding mass at the urethral meatus. These varying presentations necessitate a high level of clinical suspicion and a comprehensive diagnostic strategy.

Diagnostic delays are common in the reviewed cases, as evidenced by 44% (7/16) of cases initially misdiagnosed as vulvar cysts or pelvic organ prolapse. Ultrasound remains the first-line imaging modality because it is non-invasive, accessible, repeatable, and capable of demonstrating the cystic nature of the lesion, its continuity with the distal ureter/bladder region, and associated hydronephrosis or ureteral dilatation (3). In the present case, multimodal ultrasonography was especially valuable because it dynamically demonstrated the relationship between the prolapsed lesion, urethra, and sling, which is difficult to appreciate on routine examination alone. Compared with previously reported cases, the present case is notable for the postoperative diagnostic complexity after MUS surgery and for the use of combined TBHU and SVG to dynamically delineate the dumbbell configuration and its relationship to the sling.

Additional imaging techniques also contribute significantly to diagnostic accuracy. CT and MRI provide complementary information: CT is useful for depicting stones, upper urinary tract dilatation, and overall pelvic anatomy (27), whereas MRI offers excellent soft-tissue contrast. However, both CT and MRI are relatively static examinations and may be less effective than targeted multimodal ultrasonography for demonstrating dynamic prolapse, real-time compressibility, and the precise spatial relationship between the ureterocele, urethra, and sling (28, 29). In particular, TBHU offers high-resolution, near-field visualization of the periurethral region and sling (30), while SVG can improve delineation of the vaginal wall and prolapsed sac, thereby helping distinguish prolapsed ureterocele from urethral diverticulum, paraurethral cyst, or pelvic organ prolapse (31, 32). These techniques also facilitate recognition of the dumbbell sign by showing two communicating cystic components separated by a narrowed segment at the urethral outlet. Ultrasonography also avoids ionizing radiation, is lower cost, and can be performed repeatedly at the bedside, although its performance is operator-dependent and image quality may be reduced by patient habitus or limited acoustic windows. Cystoscopy remains an important adjunct for confirming the diagnosis and guiding treatment (33, 34).

Given the overlapping symptoms, accurate diagnosis of prolapsed ureterocele requires careful differentiation from other urogenital conditions. POP, for instance, may present with similar features. Postmenopausal patients with advanced uterine prolapse can develop bilateral hydroureteronephrosis due to ureteral compression, which may mimic ureterocele-related obstruction (35). However, ureterocele primarily involves cystic dilation of the distal ureter, whereas POP typically entails descent of the pelvic organs. Other differential considerations include vaginal or vulvar masses (3) and bladder tumors or calculi (36), especially in patients presenting with hematuria or irritative lower urinary tract symptoms.

### 5.3 Management implications and treatment of prolapsed ureterocele

The management of prolapsed ureterocele in adult females requires a comprehensive and individualized approach, taking into account the complexity of the condition and the potential for complications such as urinary obstruction, infection, or the presence of associated urological anomalies. Multidisciplinary collaboration among urologists, gynecologists, radiologists, and pelvic floor specialists is often important for treatment planning and preservation of renal function.

Endoscopic intervention is widely used for the treatment of prolapsed ureterocele due to its minimally invasive nature, reduced recovery time, and overall safety profile. Techniques such as transurethral incision, puncture, or unroofing of the ureterocele under cystoscopic guidance are commonly employed to decompress the ureterocele, facilitate urinary drainage, and relieve obstruction (37, 38).

When endoscopic methods are inadequate or contraindicated, open surgical intervention may become necessary. Ureteral reimplantation, with or without reconstruction, is a standard option for definitive management. Techniques such as the Boari flap or psoas hitch may be utilized to bridge ureteral defects and reposition the ureter in a more functional orientation (39, 40). In some cases, endoscopic decompression may be performed initially to relieve obstruction and stabilize the patient, followed by staged reconstructive surgery to address structural abnormalities and prevent recurrence (16).

The broader implications extend beyond technical refinement. First, preoperative screening for ureteroceles may be considered in MUS candidates with recurrent UTIs or congenital renal anomalies, utilizing ultrasound to identify at-risk anatomy. Second, post-MUS surveillance protocols should include prompt imaging evaluation when a vulvar mass develops, as incarceration can evolve rapidly. Finally, multidisciplinary collaboration remains important, and findings from TBHU and SVG should be interpreted together with clinical examination, other imaging studies, and cystoscopy when needed. For instance, prolapsed ureteroceles mimicking urethral diverticula on MRI may sometimes be distinguished by SVG's dynamic demonstration of urine efflux from the ureteral orifice within the prolapsed sac.

Several limitations of this systematic review should be acknowledged. First, the available evidence consists almost entirely of case reports, which are inherently prone to publication bias, as unusual presentations or favorable outcomes are more likely to be reported. Consequently, the pooled descriptive findings in this review should be regarded as hypothesis-generating rather than definitive. Second, the small sample size (16 cases) precludes robust statistical analysis and limits the generalizability of our findings. Third, substantial heterogeneity exists across the included studies with respect to diagnostic criteria, imaging work-up, treatment strategies, and reporting quality, introducing information bias and complicating direct comparison between cases. This heterogeneity is particularly evident in imaging modalities, which ranged from intravenous pyelography in earlier reports to multimodal ultrasonography and MRI in more recent cases. These differences limit the interpretability of pooled percentages and reduce the generalizability of the observed patterns. Fourth, because all included studies were retrospective in nature, they are susceptible to selection bias and incomplete data collection, with some early reports lacking detailed clinical characteristics or follow-up information. Fifth, the absence of a control group prevents assessment of causal relationships, particularly regarding whether MUS surgery may contribute to ureterocele prolapse or incarceration. Finally, although the JBI appraisal suggested generally acceptable reporting completeness, this tool cannot overcome the inherent methodological limitations of case-report evidence. In addition, while multimodal ultrasonography was highly informative in the present case, its broader diagnostic performance still requires validation across centers. Follow-up durations were variably reported and were often short, which limits assessment of long-term recurrence and functional outcomes; similarly, follow-up in the present case was limited to three months. Therefore, these findings should be interpreted with caution, and larger prospective registries or collaborative studies are needed to better characterize this rare condition and inform evidence-based management.

## 6 Conclusion

IPU following MUS surgery represents an exceptionally rare yet clinically important complication in adult women. This case highlights the importance of careful differential diagnosis when evaluating postoperative urinary symptoms, particularly when atypical presentations—such as a prolapsed ureterocele—may mimic more common urogenital disorders. The use of multimodal ultrasonography, including TBHU and SVG, was helpful in establishing an accurate and timely diagnosis. These tools improved visualization of the pelvic floor and periurethral structures, facilitating the differentiation of prolapsed ureterocele from other pathologies. Timely surgical management, encompassing cystoscopic resection of the ureterocele and modification of the MUS sling, was associated with a favorable short-term outcome in the present case. Given the extreme rarity of this condition, it is essential to raise clinical awareness among gynecologists, urologists, and pelvic floor specialists. Early recognition and multidisciplinary collaboration may help improve diagnosis and management in patients with this rare condition.
