# Correction: Incarcerated prolapsed ureterocele masquerading as a vulvar mass after midurethral sling surgery: a case report with systematic review

**DOI:** 10.3389/fsurg.2026.1916579

**Published:** 2026-07-06

**Authors:** Hongyun Zhang, Jiang Zhu, Zihan Zhou, Xia Tao, Liqun Sun, Hong Zhan

**Affiliations:** 1 Department of Ultrasound, Women’s Hospital, Zhejiang University School of Medicine, Hangzhou, China; 2 Zhejiang University School of Medicine, Hangzhou, China; 3 Day Surgery Center, Women’s Hospital, Zhejiang University School of Medicine, Hangzhou, China; 4 Zhejiang Provincial Key Laboratory of Precision Diagnosis and Therapy for Major Gynecological Diseases, Women’s Hospital, Zhejiang University School of Medicine, Hangzhou, China

**Keywords:** midurethral slings, pelvic organ prolapse, sonovaginography, ultrasonography, ureterocele

Author Hongyun Zhang was erroneously assigned as corresponding author. The correct corresponding author is Hong Zhan.

The original version of this article has been updated.

