# Supplementary material for: Incarcerated prolapsed ureterocele masquerading as a vulvar mass after midurethral sling surgery: a case report with systematic review
Source: Front Surg. 2026 Jun 9;13:1773513. doi: 10.3389/fsurg.2026.1773513 (PMC13287005; doi:10.3389/fsurg.2026.1773513)
Supplement: Supplementary file 1 [file Table1.docx]

**Supplementary Table 1. Quality appraisal of the included case reports using the Joanna Briggs Institute checklist**

| **Case (Reference)** | **Q1** | **Q2** | **Q3** | **Q4** | **Q5** | **Q6** | **Q7** | **Q8** | **Total** |
| --- | --- | --- | --- | --- | --- | --- | --- | --- | --- |
| P1 | Y | Y | Y | Y | Y | Y | Y | Y | 8/8 |
| P2(17) | Y | Y | Y | Y | Y | Y | Y | Y | 8/8 |
| P3(15) | Y | Y | Y | Y | Y | Y | Y | Y | 8/8 |
| P4(16) | Y | Y | Y | Y | Y | Y | Y | Y | 8/8 |
| P5(3) | Y | Y | Y | Y | Y | Y | N | Y | 7/8 |
| P6(20) | Y | Y | Y | Y | Y | Y | Y | Y | 8/8 |
| P7(11) | Y | Y | Y | Y | Y | N | Y | N | 6/8 |
| P8(7) | Y | Y | Y | Y | Y | N | Y | Y | 7/8 |
| P9(13) | Y | N | Y | Y | Y | Y | Y | Y | 7/8 |
| P10(8) | Y | Y | Y | Y | Y | Y | Y | Y | 8/8 |
| P11(10) | Y | Y | Y | Y | Y | N | N | Y | 6/8 |
| P12(12) | Y | N | Y | Y | Y | Y | Y | Y | 7/8 |
| P13(19) | Y | Y | Y | Y | Y | N | Y | Y | 7/8 |
| P14(18) | Y | Y | Y | Y | Y | Y | Y | N | 7/8 |
| P15(14) | Y | Y | Y | Y | Y | Y | Y | Y | 8/8 |
| P16(9) | Y | Y | Y | Y | Y | N | Y | N | 6/8 |

**Abbreviations:** JBI, Joanna Briggs Institute; Y, yes; N, no.

**JBI questions:** Q1, patient demographic characteristics; Q2, patient history presented as a timeline; Q3, current clinical condition on presentation; Q4, diagnostic tests or assessment methods and results; Q5, intervention or treatment procedure; Q6, post-intervention clinical condition; Q7, adverse events or unanticipated events identified and described; Q8, takeaway lessons.

**Note:** The JBI checklist was used to appraise reporting completeness of the included case reports. No overall risk-of-bias category was assigned because this tool is not designed to generate a validated overall bias grade for case reports. Cases were labeled according to the final reference list in the manuscript; the present patient is shown separately.
